# Biomechanical Response of Lung Epithelial Cells to Iron Oxide and Titanium Dioxide Nanoparticles

**DOI:** 10.3389/fphys.2019.01047

**Published:** 2019-08-16

**Authors:** Vinícius Rosa Oliveira, Juan José Uriarte, Bryan Falcones, Ignasi Jorba, Walter Araujo Zin, Ramon Farré, Daniel Navajas, Isaac Almendros

**Affiliations:** ^1^Unitat de Biofísica i Bioenginyeria, Facultat de Medicina, Universitat de Barcelona, Barcelona, Spain; ^2^Laboratório de Fisiologia da Respiração, Instituto de Biofísica Carlos Chagas Filho, Universidade Federal do Rio de Janeiro, Rio de Janeiro, Brazil; ^3^Institute for Bioengineering of Catalonia, The Barcelona Institute of Science and Technology, Barcelona, Spain; ^4^Centro de Investigación Biomédica en Red Enfermedades Respiratorias (CIBERES), Madrid, Spain; ^5^Institut d’Investigacions Biomèdiques August Pi i Sunyer (IDIBAPS), Barcelona, Spain

**Keywords:** air pollution, lung epithelium, cell biomechanics, nanoparticles, actomyosin fibers

## Abstract

Increasing evidence shows that lungs can be damaged by inhalation of nanoparticles (NPs) at environmental and occupational settings. Recent findings have associated the exposure to iron oxide (Fe_2_O_3_) and titanium dioxide (TiO_2_) – NPs widely used in biomedical and clinical research – with pulmonary oxidative stress and inflammation. Although changes on cellular mechanics could contribute to pulmonary inflammation, there is no information regarding the effects of Fe_2_O_3_ and TiO_2_ on alveolar epithelial cell biomechanics. The aim was to investigate the NPs-induced biomechanical effects in terms of cell stiffness and traction forces exerted by human alveolar epithelial cells. Cell Young’s modulus (*E*) measured by atomic force microscopy in alveolar epithelial cells significantly decreased after exposure to Fe_2_O_3_ and TiO_2_ (∼28 and ∼25%, respectively) compared to control conditions. Moreover, both NPs induced a similar reduction in the traction forces exerted by the alveolar epithelial cells in comparison to the control conditions. Accordingly, immunofluorescence images revealed a reduction of actomyosin stress fibers in response to the exposure to NPs. However, no inflammatory response was detected. In conclusion, an acute exposure of epithelial pulmonary cells to Fe_2_O_3_ and TiO_2_ NPs, which was mild since it was non-cytotoxic and did not induce inflammation, modified cell biomechanical properties which could be translated into damage of the epithelial barrier integrity, suggesting that mild environmental inhalation of Fe_2_O_3_ and TiO_2_ NPs could not be innocuous.

## Introduction

Nanoparticles (NPs) constitute a fraction of atmospheric particulate matter (PM) and are defined as ultrafine structures because of their less than 100 nm diameter (Donaldson et al., 2001). Environmental pollution is a major health concern, especially in developing countries, with reported levels of PM 10 times higher than in developed countries (Lee et al., 2014). The most frequent scenarios of PM exposure are environmental and occupational settings (factories and coal mines). Epidemiological studies show that exposure to airborne PM is closely associated with increased morbidity and mortality from respiratory and cardiovascular diseases, accounting to 3.2 million deaths per year (Samet et al., 2000; Weichenthal et al., 2007; Lim et al., 2012). The prominent respiratory effects associated to the exposure to airborne PM are lung inflammation and papillary adenocarcinoma (Yamadori et al., 1986; Andujar et al., 2014).

According to their classification, iron oxide (Fe_2_O_3_) and titanium dioxide (TiO_2_) are inorganic materials of natural (geogenic) and anthropogenic (engineered) sources, respectively (Nowack and Bucheli, 2007). During the last decade, these NPs have attracted considerable attention for application in biomedical and clinical research because their unique physicochemical properties (Huber, 2005; McCarthy and Weissleder, 2008; Yin et al., 2013). Titanium dioxide also is used as an ingredient in many pharmaceutical products and cosmetics (Grande and Tucci, 2016). Increasing evidence shows that both Fe_2_O_3_ and TiO_2_ NPs can promote lung inflammation and remodeling of the pulmonary parenchyma, contributing to fibrosis and granuloma formation (Yamadori et al., 1986; Hwang et al., 2010; Ban et al., 2013; Kim et al., 2017). At the cellular level, Fe_2_O_3_ and TiO_2_ can increase the production of reactive oxygen species (ROS), which has been linked to cell architecture motility and DNA alterations (Ghosh et al., 2010; Zhu et al., 2010; Hanot-Roy et al., 2016). Considering that their impact on human health is predictably increasing, these NPs are being extensively evaluated in terms of their health effects on the lungs via oxidative stress.

Injurious physical (hyperoxia, over-stretch) and chemical (endotoxin, thrombin) stimuli can induce actin reorganization in alveolar epithelial cells which has been associated with changes in cell rheology (Trepat et al., 2004; Gavara et al., 2006; Byfield et al., 2011; Puig et al., 2013; Wilhelm et al., 2014). Furthermore, an increase in cell stiffness has been demonstrated that could facilitate the disruption of the alveolar–capillary barrier in alveolar epithelial cells (Puig et al., 2013). Our hypothesis is that Fe_2_O_3_ and TiO_2_ NPs could also induce changes in the biomechanical properties of epithelial lung cells explaining in part the inflammatory effects of these NPs observed *in vivo*.

Considering that alveolar epithelial cells are the first barrier encountered by NPs once inhaled, this study was carried out with a well characterized human alveolar epithelial-like cell line exposed to either Fe_2_O_3_ or TiO_2_ NPs. The effects of this challenge on cell biomechanics, specifically cell stiffness and cell force generation on the substrate, were measured by atomic force microscopy (AFM) and traction microscopy.

## Materials and Methods

### Cell Culture

The study was carried out on A549 alveolar epithelial cells (ATCC^®^ CCL-185), which were cultured in RPMI-1640 medium supplemented with 10% fetal bovine serum (FBS), 1% penicillin, streptomycin and amphotericin B, 10 mM HEPES and 1 mM glutamine (Sigma-Aldrich, St. Louis, MO, United States). Cells were maintained in an incubator with humidified environment containing 5% CO_2_ at 37°C and pH 7.4. Cell culture and reagent handling were carried out in agreement with the University of Barcelona Biosecurity Committee guidelines.

### Fe_2_O_3_ and TiO_2_ Nanoparticles Preparation and Characterization

Iron oxide (Fe_2_O_3_, #544884) and titanium dioxide (TiO_2_, #718467) nanopowder were purchased from Sigma-Aldrich (St. Louis, MO, United States). Stock solutions of Fe_2_O_3_ and TiO_2_ NPs were prepared in ultrapure water (1 mg/mL), sonicated using an ultrasonic homogenizer (BANDELIN Electronic GmbH & Co. KG, Berlin, Germany) operating at 4 W for 5 min, and diluted to 10, 50, 100, and 200 μg/mL in serum-free medium.

Dynamic light scattering (DLS) was used to determine the size of NPs aggregates and their agglomeration status. Fe_2_O_3_ and TiO_2_ suspensions (50 μg/mL) were prepared in culture medium and measured with a Zetasizer Nano ZS (Malvern Instruments, Malvern, United Kingdom) in single replicates performed on three unique days. Alternatively, transmission electron microscopy (TEM) was employed at an accelerating voltage of 200 kV (JEM-2100Plus, Tokyo, Japan). Particle suspensions were prepared in 100% ethanol, placed onto standard carbon-coated copper grids and then air-dried at room temperature before images were processed.

### Cell Internalization of Nanoparticles

Transmission electron microscopy was also used to study the potential ultrastructural modifications induced in cells and to visualize NPs in the intracellular compartment. Cell ultrastructure was observed after 24 h-exposure to 10 μg/mL of Fe_2_O_3_ or TiO_2_ NPs, respectively in single replicates performed on three unique days. Cell monolayers were fixed in 2.5% glutaraldehyde in sodium phosphate buffer (Na_2_HPO_4_ × 2H_2_O) and post-fixed with 2% paraformaldehyde. Ultrathin sections of 60 nm were cut on an ultramicrotome and then observed with a JEOL 1010 (JEOL USA Inc., Peabody, MA, United States) transmission electron microscope, equipped with an Orius^®^ CCD camera (Gatan Inc., Pleasanton, CA, United States), operating at an accelerating voltage of 80 kV.

### Cell Cytotoxicity

Lactate dehydrogenase (LDH) activity was measured using the Cytotoxicity Detection Kit^PLUS^ (version 6, Roche, Basel, Switzerland) following the manufacturer’s instructions. Briefly, cells were seeded into 96 multi-well plates (1 × 10^4^ cells/well) and treated for 24 h with different concentrations of Fe_2_O_3_ and TiO_2_ NPs (10, 50, 100, and 200 μg/mL). The percentage of cytotoxicity was calculated using the mean of three replicates from three independent experiments performed on different days according to the following equation:

$$\text{Cytotoxicity}\left(\%\right)=\frac{\mathrm{Experimental}\,\mathrm{value}-\mathrm{Low}\,\mathrm{control}}{\mathrm{High}\,\mathrm{control}-\mathrm{Low}\,\mathrm{control}}\times 100$$

### Measurement of Cell Stiffness

Cells were cultured until 60% confluence (24–48 h) and then were exposed to either Fe_2_O_3_, TiO_2_ NPs (10 μg/mL) or culture medium for 24 h. Cell stiffness was measured by a custom-built AFM from 11 independent experiments repeated in different days, 1 sample per group (12 cells/sample) as described previously (Alcaraz et al., 2003). Force-indentation curves were obtained with a V-shaped Au-coated cantilever (spring constant = 0.03 N/m) with a spherical tip (4.5 μm-diameter) on its apex (Novascan Technologies, Inc., Boone, IA, United States). The cantilever was placed on the perinuclear region of 12 cells from each sample. Each measurement consisted of five force–displacement curves (*F*–*z*) (triangular ramp, 1 Hz oscillation, 4 μm peak-to-peak ramp amplitude, and a maximum indentation of 1000 nm). Young’s modulus (*E*) was computed by fitting the *F*–*z* curve with the Hertz contact model (Alcaraz et al., 2003).

$$F=\frac{4\,E}{3\,\left(1-\upsilon^{2}\right)}\,\sqrt{R}\,\delta^{\frac{3}{2}}$$

where *F* = force, *E* = Young’s modulus, υ = Poisson’s ratio (typically 0.5), *R* = radius of the indenter (2250 nm), and δ = indentation (up to 1000 nm).

### Cell Traction Measurements

Cells were seeded at 40% confluence and cell traction forces were determined by traction force microscopy (TFM; Gavara et al., 2006). Cells were then exposed to Fe_2_O_3_ and TiO_2_ NPs (10 μg/mL) or serum-free medium for 24 h. The samples (*n* = 7 experiments per group repeated in different days, 7–8 cells/sample) were placed on a microscope (Eclipse Ti, Nikon Instruments, Amsterdam, Netherlands) equipped with a CCD camera (C9100, Hamamatsu Photonics K.K., Hamamatsu, Japan) to measure cell forces. For each traction field, the total force magnitude was computed by integrating the magnitude of the traction field over the projected area of the cell.

### Inflammatory Cytokine Assessment

In a separate series of experiments (*n* = 6 rats per group), the content of IL-1β and IL-8 released by cells in response to Fe_2_O_3_ and TiO_2_ NPs (10 μg/mL) were determined by ELISA (Quantikine ELISA Kit; R&D Systems, Minneapolis, MN, United States) following the manufacturer’s instructions.

### Immunofluorescence Staining of Actin Stress Fibers

Measurement of actin stress fiber formation was carried out using a single sample per group from six independent experiments carried out in different days. Cells were cultured on 18 mm-diameter coverslips (Knittel Glässer, Braunschweig, Germany). Once cells reached to 70% confluence, were exposed to either NPs (10 μg/mL Fe_2_O_3_ or 10 μg/mL TiO_2_) or culture medium for 24 h. Then, cells were washed 3X with PBS, fixed with 4% formaldehyde-PBS solution for 15 min at room temperature and washed 3X with PBS. Afterward F-actin was stained with a conjugate Alexa Fluor 555-phalloidin (A33405, Thermo Fisher Scientific, MA, United States) according to the manufacturer’s instructions. Briefly, cells were permeabilized with 0.1% Triton X-100 for 5 min and washed with PBS. Then, cells were incubated with phalloidin fluorescent conjugate in a solution of 1% of BSA in PBS for 45 min, washed 3X with PBS for 5 min and mounted after coverslip drought onto immuno slides with a drop of Fluoromount (SouthernBiotech, AL, United States). Imaging was performed with a confocal laser scanning microscope (Eclipse Ti, Nikon Instruments, Amsterdam, Netherlands) at 20× magnification equipped with a Nikon confocal (D-eclipse C1, Nikon Instruments, Amsterdam, Netherlands). The percentage of cells presenting peripheral stress fibers was quantified (from each sample an image of the central region of the coverslip was taken and more than 350 cells were counted). Cell staining, as well as stress fiber quantification, were carried out under experimental blind conditions.

### Statistics

One-way ANOVA analysis followed by Student–Newman–Keuls test was used to assess differences among groups (SigmaPlot 11 statistical package, SYSTAT Software, Chicago, IL, United States). All values are presented as mean ± SE.

## Results

### NPs Characterization and Cell Internalization

Dynamic light scattering measurements revealed that Fe_2_O_3_ tended to form 1.5-fold larger aggregates than TiO_2_ (average diameter of 602.3 and 383.7 nm, respectively), Table 1 and Supplementary Material. This analysis also showed heterogeneous agglomerates with considerable polydispersity in both types of NPs.

**TABLE 1:** Physicochemical properties of Fe_2_O_3_ and TiO_2_ nanoparticles.

	**Fe_2_O_3_**	**TiO_2_**
*Data from manufacturer*		
Nominal size (nm)	<50	21
Surface area (m^2^/g)	50–245	35–65
*DLS analyses*		
Mean diameter (nm)	602.3 ± 9.87	383.7 ± 16.68
PDI	0.52 ± 0.01	0.40 ± 0.03

*Characterization of Fe_2_O_3_ and TiO_2_ suspensions (10 μg/mL) in culture medium. Values are mean ± SEM of three determinations. DLS, dynamic light scattering; PDI, polydispersity index.*

**FIGURE 1:**
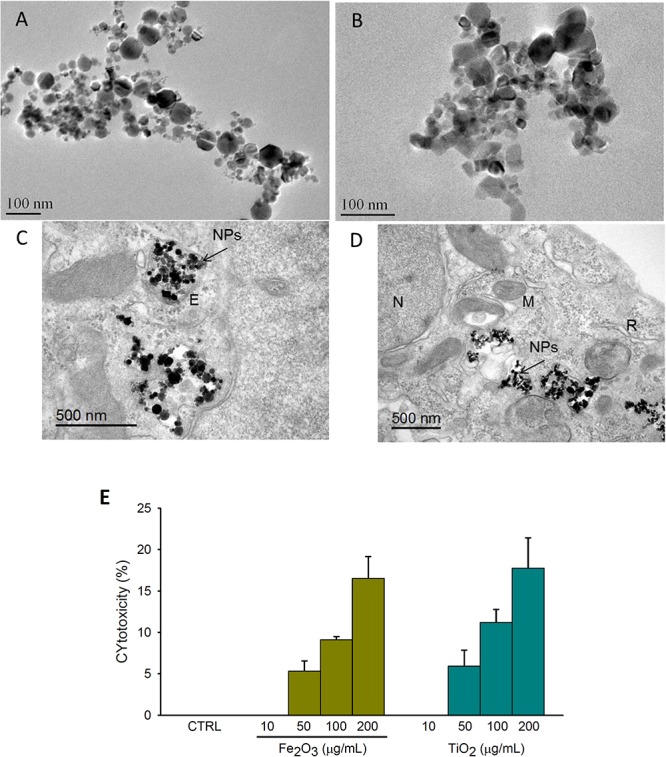
Nanoparticles characterization, internalization and cell viability. Transmission electron microscopy (TEM) images of: **(A,B)** Fe_2_O_3_ and TiO_2_ aggregates, respectively; **(C,D)** A549 cell ultrastructure after 24 h-exposure to 10 μg/mL of Fe_2_O_3_ and TiO_2_ NPs, respectively. Note the presence of NPs aggregates trapped in vesicles **(C)** and also spread in the cytoplasm **(D)**. Lactate dehydrogenase (LDH) release expressed as percentage of cytotoxicity after 24 h-exposure to 10, 50, 100, and 200 μg/mL of Fe_2_O_3_ and TiO_2_ NPs or culture medium (CTRL) in A549 **(E)**. E, endosome; M, mitochondria; N, nucleus; R, rough endoplasmic reticulum. Data are presented as mean ± SE, *n* = 3.

TEM imaging (Figure 1) show that, similarly to DLS, both NPs were predominantly presented in aggregates but presenting different shape type. Fe_2_O_3_ aggregates had mainly hexahedron shape whereas TiO_2_ presented irregular but nearly round shape. Cell uptake of NPs was also confirmed by TEM images (Figure 1). Specifically, NPs were found within the cytoplasm and also trapped in vesicles indicating phagocytosis and/or micropinocytosis as a potential internalization mechanism.

### Cytotoxic and Inflammatory Effects of NPs on Pulmonary Cells

Lactate dehydrogenase release, expressed as percentage of cytotoxicity, showed no cytotoxic effects for NPs concentrations ≤10 μg/ml. For higher concentrations of Fe_2_O_3_ and TiO_2_ NPs, a dose-dependent profile was observed (Figure 1).

Remarkably, no significant changes in the expression of IL-8 were found between groups (*p* = 0.149). IL-1β protein were not detected in any group.

### Exposure to NPs Reduce Lung Epithelial Cell Stiffness, Traction Forces, and F-Actin Stress Fibers

**FIGURE 2:**
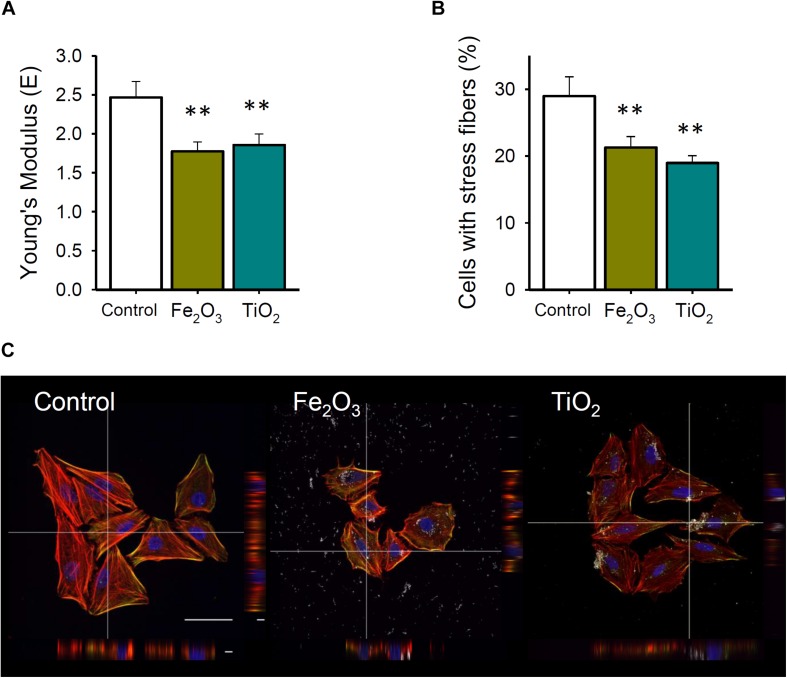
**(A)** Cell stiffness after 24 h exposure to Fe_2_O_3_ and TiO_2_ (10 μg/mL) NPs or culture medium (CTRL) (*n* = 11). **(B)** Percentage of cells presenting actomyosin stress fibers after 24 h of exposure to Fe_2_O_3_ and TiO_2_ (10 μg/mL) NPs or CTRL (*n* = 6). **(C)** Examples of F-actin (red) and myosin light chain (green) stained cells. Data are presented as mean ± SE. ^∗∗^*P* < 0.01.

Cell stiffness measurements performed for exposure to 10 μg/mL Fe_2_O_3_ or TiO_2_ NPs as well as in control conditions are shown in Figure 2. Fe_2_O_3_ and TiO_2_ NPs significantly decreased cell stiffness (1.77 ± 0.12 Pa; *p* = 0.012 and 1.86 ± 0.14 Pa; *p* = 0.011, respectively) respect to control conditions (2.46 ± 0.20 Pa).

**FIGURE 3:**
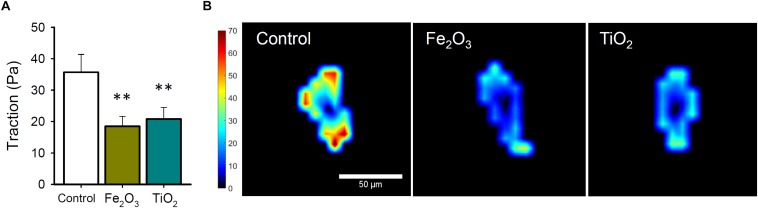
**(A)** Cell traction forces after 24 h-exposure to Fe_2_O_3_ and TiO_2_ (10 μg/mL) NPs or culture medium (CTRL) in A549 cells (*n* = 7). **(B)** Representative maps of cell traction forces for each experimental condition [color scale bar in Pascals (Pa)]. Data are presented as mean ± SE. ^∗∗^*P* < 0.01.

Epithelial cells exhibited significantly decreased traction forces when exposed to Fe_2_O_3_ (18.49 ± 3.18 Pa; *p* = 0.029) and TiO_2_ NPs (20.82 ± 3.65 Pa; *p* = 0.026) compared to control conditions (35.69 ± 5.69 Pa) (Figure 3).

In agreement with reduction in cell stiffness and traction forces, actin cytoskeleton was remodeled after Fe_2_O_3_ and TiO_2_ NPs exposures. In control conditions, F-actin was rearranged in thick bundles and even some cells present marked stress fibers at the cell periphery (Figure 2). Specifically, the percentage of cells presenting these actin stress fibers was 29.0 ± 2.9% which was significantly reduced to 21.3 ± 1.6% (*p* = 0.029) and 19.0 ± 1.1% (*p* = 0.026) when cells were exposed to Fe_2_O_3_ and TiO_2_ NPs, respectively.

## Discussion

The present study demonstrates that non-cytotoxic concentrations of Fe_2_O_3_ and TiO_2_ NPs promote considerable biomechanical changes in human alveolar epithelial cells even in the absence of an inflammatory response. Specifically, both types of NPs significantly reduce the stiffness and mechanical traction forces of cells which could be explained with the loss of actin stress fibers observed.

The NPs employed in this study were chosen because their increasing employment in a wide range of fields. Considering that their impact on human health is predictably increasing, these NPs are being extensively evaluated in terms of their health effects on the lungs via oxidative stress (Sadeghi et al., 2015). Fe_2_O_3_ NPs are ultrafine particles commonly found in urban atmospheric aerosols and have lately called for great interest in biomedicine research as magnetic resonance imaging (MRI) contrast agents, drug delivery carriers and cell labeling reagents (Huber, 2005; Huang et al., 2009; Tang et al., 2017). In the same line, TiO_2_ are considered the largest synthetized and broadly distributed NPs, with applications in the biomedical field (cell imaging and genetic engineering), as well as in the consumer industry (cosmetics, sunscreen, toothpaste and paints) (Oh and Park, 2011; Yin et al., 2013). Taken together, respiratory exposure to these NPs pose a concern to individuals living in urban areas with high levels of environmental PM, and occupational exposure to nanomaterials, electronic and electrical waste industries. With respect to the hazardous effects of iron oxide NPs, Lai et al. (2015) showed that different metallic NPs can result in different cell responses. They employed Fe_2_O_3_ and ZnO in two pulmonary epithelial cell lines (BEAS-2B and A549 cell lines) and found that only ZnO, but not Fe_2_O_3_, NPs induce cell cycle arrest, cell apoptosis, oxidative stress, and glucose metabolism alterations promoting cytotoxicity. However, these authors used doses ranging from 5 to 200 μg/ml for both NPs, revealing that lung epithelial cells are much more respondant to ZnO than to Fe_2_O_3_. In contrast to these findings, Sadegui et al. (2015) investigated the toxic effects of Fe_2_O_3_ NPs on lung tissue in rats. These particles at a concentration between 20 and 40 mg/kg and with several exposures (7 and 14 times) can boost the production of free radicals and reduce glutathione (GSH) expression. Furthermore, the authors found that Fe_2_O_3_ NPs can develop pulmonary emphysema, interstitial hyperemia and infiltration of inflammatory cells in the lung tissue. As in the case of Fe_2_O_3_ NPs, the toxicity of TiO_2_ NPs has been attributed to the generation and accumulation of ROS that drive inflammatory responses (Tang et al., 2013). In addition, these inflammatory effects were related to TiO_2_ NPs structural features, including size, shape, crystal phases, dispersion and agglomeration status, surface coating, and chemical composition (Wang and Fan, 2014).

Considering the current controversial data about the toxic effects of these particles, and prior to the biomechanical study, a dose-response (ranging from 10 to 200 μg/mL) cytotoxicity test was performed in response to Fe_2_O_3_ and TiO_2_ NPs to avoid any potential biomechanical change caused by cell damage. As previously reported, we found that NPs-induced cytotoxicity was dose dependent and that, for both NPs, 10 μg/mL was the highest dose not presenting cytotoxicity. In this regard, Ursini et al. (2014) found similar cytotoxic effects of TiO_2_ on A549 cells acutely exposed to 1– 40 μg/mL and cytotoxicity was associated with physical characteristics (size and crystal phase) (Moschini et al., 2013). In addition, and considering the aforementioned importance of NPs structure on their cytotoxicity (Wang and Fan, 2014), both NPs were morphologically characterized by DLS and TEM prior to experiments. The average diameter and polydispersion of these NPs dissolved in medium were in accordance with previous studies (Cohen et al., 2013). Concerning cell uptake, TEM images revealed cytosolic clusters of Fe_2_O_3_ and TiO_2_ NPs after a few hours of exposition at a concentration of 10 μg/mL. These clusters of NPs had different sizes as previously observed by others (Apopa et al., 2009; Ahlinder et al., 2013; Moschini et al., 2013). In addition, the lack of increased expression in two relevant inflammatory cytokines (Il-1β and IL-1), suggest the dose employed with both NPs were innocuous. Nevertheless, and more interestingly, the biomechanical changes reported here in response to NPs are even earlier than the expression of these cytokines.

Atomic force microscopy is becoming a prevalent tool in biomedicine and has been widely used to study the mechanical properties of resting living cells or in response to different chemical or physical stimuli. AFM, among other biophysical techniques, have been employed to explain how mechanical forces exerted by epithelial cells in response to inflammatory and other cytotoxic agents can contribute in the alveolar epithelial-barrier disruption (Trepat et al., 2004; Gavara et al., 2006; Byfield et al., 2011; Puig et al., 2013; Wilhelm et al., 2014; Oliveira et al., 2019). The changes in the Young’s modulus of the cells have been postulated to be produced by multiple causes, including cytoskeleton rearrangements, modifications on the cell surface and/or changes in the protein content (myosin and F-actin/G-actin ratio) (Gavara et al., 2008; Ogneva et al., 2014). The available studies aiming to address how NPs can induce changes in the biomechanical properties of pulmonary epithelial cells are still very scarce. Furthermore, these studies only cover a very limited number of environmental NPs whose exposure and uptake into pulmonary epithelial cells could have different effects on cell rheology. In this work, we found that a typical model of alveolar epithelial cell exposed to Fe_2_O_3_ and TiO_2_ NPs presented a reduction in cell stiffness. From previous studies, Subbiah et al. (2015) reported an increase in cell stiffness after exposure to silver NPs in several cell types including A549 cells, human bone marrow stromal cells (H-S5) and breast cancer cells (NIH3T3). However, the dose employed by these authors was the highest tested here (40 μg/mL of NPs), which presented clear cytotoxic effects on all the three types of cells used. Besides, Pi et al. (2013) showed a similar decrease of Young’s modulus of breast cancer cells (MCF-7) exposed to selenium NPs. In accordance with our results, these authors linked the decrease of Young’s modulus observed in MCF-7 cells to the dis-organization and down-regulation of F-actin induced by selenium NPs. Specifically, at control conditions A549 cells displayed more fluorescent intensity for myosin light chain 2 than NPs-exposed cells. Although these alterations seemed rather slight, the aforementioned cell lines exhibited significant biomechanical changes that are possibly linked to unfolding cytoskeleton network. Stress fibers were mainly observed at the lowermost region of the cells, close to the substrate, whereas vesicles containing NPs localized mostly at the cortical perinuclear area. We speculate that the decreased stiffness observed in A549 cells could as well be associated with the decrease in cytoskeleton tension elicited by changes in actin polymerization. However, we cannot discard the contribution of other components not evaluated in this study, such as intermediate filaments and microtubules.

Cellular tension is predominantly generated by non-muscle myosin II, which crosslinks actin filaments when activated, producing effects on contraction, morphological stability, motility and regulation of focal adhesions (Martens and Radmacher, 2008). AFM and F-actin polymerization analyses suggested a potential reduction in the traction forces exerted by A549 cells exposed to NPs. TFM is a technique that allows assessing the regional distribution of contractile forces at the single-cell level and the understanding of cell-extracellular matrix interactions. Herein, we observed a significant reduction in the traction forces exerted by A549 after NPs exposure. These findings could be explained by interplay between the diminished centripetal forces from the cytoskeleton and a possible weakening of cell adhesions (Gavara et al., 2006). We could hypothesize that exposure to NPs promoted a degree of cell detachment that caused decrease in stiffness and, thus yielded the observed effects.

In conclusion, an acute non-cytotoxic exposure of epithelial pulmonary cells to NPs is enough to alter the biomechanical properties of these cells. The reduced stiffness and diminished traction forces after exposure to Fe_2_O_3_ and TiO_2_ NPs could be reflected on changes in epithelial permeability or could also modulate the barrier integrity alterations induced by other physical or chemical injurious challenges. It is worth mentioning that adenocarcinoma human alveolar epithelial cells were used in this study. Despite being largely used *in vitro* to reproduce the effects of noxious agents in the respiratory system, more studies should be conducted with different cell lines and/or primary culture to confirm the potential damage caused by NPs. Thus, considering the public health drawbacks arising from NPs exposure, NPs are not as innocuous as initially though and their exposure during the work practice and engineering control procedures needs to be prevented. In this connection, the results in this study suggest that cell stiffness could be a very early biomarker of cell damage by NPs.

## Author Contributions

DN, IA, RF, VO, and WZ conceived the study. VO, JU, IJ, and BF performed the experiments. VO, RF, DN, and IA contributed to the study design, data analysis, and discussions along with the project. VO and IA drafted the manuscript. All authors have read and approved the final draft of the manuscript.

## Conflict of Interest Statement

The authors declare that the research was conducted in the absence of any commercial or financial relationships that could be construed as a potential conflict of interest.
