# Supplementary material for: Biomechanical Response of Lung Epithelial Cells to Iron Oxide and Titanium Dioxide Nanoparticles
Source: Front Physiol. 2019 Aug 16;10:1047. doi: 10.3389/fphys.2019.01047 (PMC6707084; doi:10.3389/fphys.2019.01047)
Supplement: Supplementary file 1 [file Data_Sheet_1.PDF]

Supplementary Material

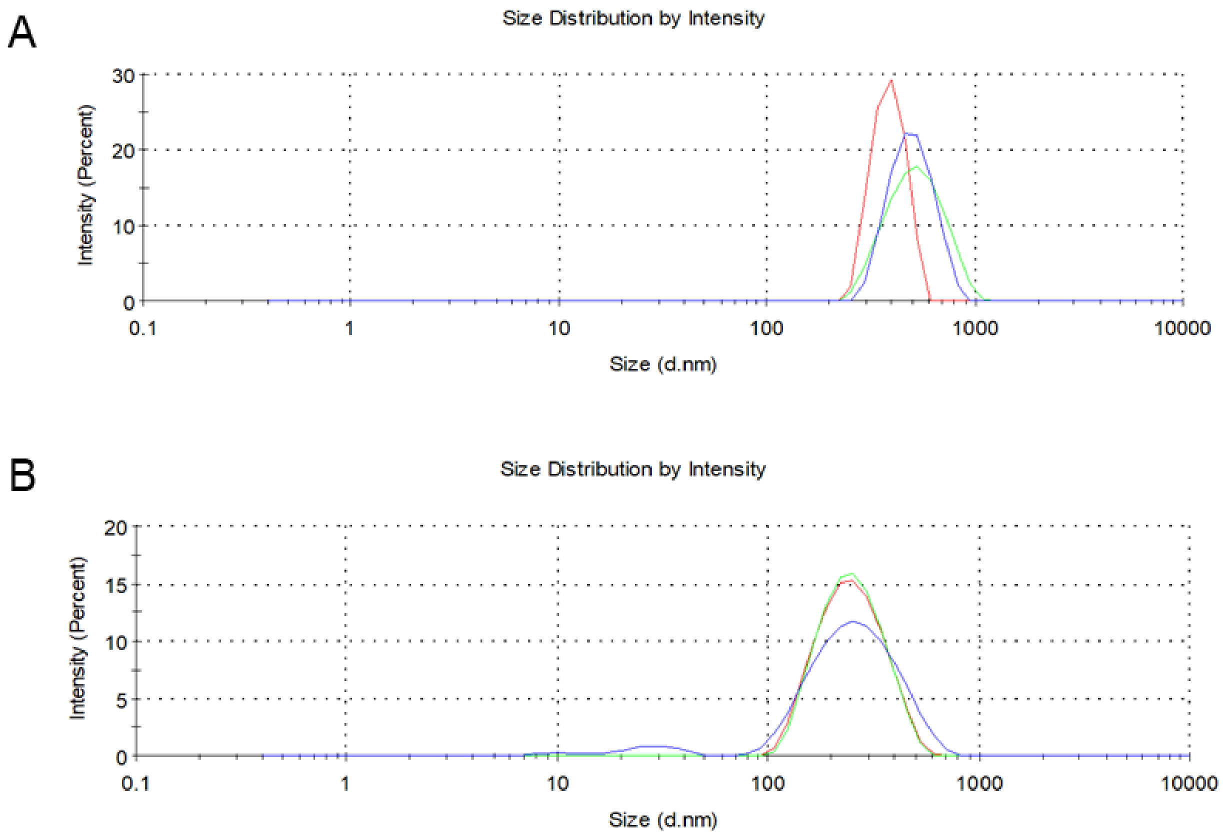

Supplementary Figure 1. Dynamic Light Scattering size (diameter: d) distribution by intensity of Fe_2O_3 (A) and TiO_2 NPs (B) (each curve represents an independent experiment, n=3).
